# CXCL13 and Its Receptor CXCR5 in Cancer: Inflammation, Immune Response, and Beyond

**DOI:** 10.3389/fendo.2019.00471

**Published:** 2019-07-12

**Authors:** Marcelo G. Kazanietz, Michael Durando, Mariana Cooke

**Affiliations:** Department of Systems Pharmacology and Translational Therapeutics, Perelman School of Medicine, University of Pennsylvania, Philadelphia, PA, United States

**Keywords:** CXCL13, CXCR5, inflammation, immune responses, cancer progression

## Abstract

It is well-established that the chemokine C-X-C motif ligand 13 (CXCL13) and its receptor, the G-protein coupled receptor (GPCR) CXCR5, play fundamental roles in inflammatory, infectious and immune responses. Originally identified as a B-cell chemoattractant, CXCL13 exerts important functions in lymphoid neogenesis, and has been widely implicated in the pathogenesis of a number of autoimmune diseases and inflammatory conditions, as well as in lymphoproliferative disorders. Current evidence also indicates that the CXCL13:CXCR5 axis orchestrates cell-cell interactions that regulate lymphocyte infiltration within the tumor microenvironment, thereby determining responsiveness to cytotoxic and immune-targeted therapies. In this review, we provide a comprehensive perspective of the involvement of CXCL13 and its receptor in cancer progression. Studies in recent years postulated novel roles for this chemokine in controlling the cancer cell phenotype, and suggest important functions in the growth and metastatic dissemination of solid tumors. Carcinogens have been found to induce CXCL13 production, and production of this chemokine within the tumor milieu has been shown to impact the proliferation, migration, and invasive properties of cancer cells. Thus, the complex networks of cellular interactions involving tumoral CXCL13 and CXCR5 integrate to promote cancer cell autonomous and non-autonomous responses, highlighting the relevance of autocrine and paracrine interactions in dictating the cancer phenotype. Dissecting the molecular and signaling events regulated by CXCL13 and how this chemokine dynamically controls the interaction between the cancer cell and the tumor microenvironment is key to identify novel effectors and therapeutic targets for cancer treatment.

## Introduction

Chemokines are a family of small molecular weight proteins known for their ability to act as chemoattractants, thereby functioning to induce the migration of nearby responding cells. These secreted proteins, together with a host of other extracellular mediators, including growth factors and eicosanoids, are key modulators of inflammation by controlling complex interaction networks via autocrine and paracrine mechanisms. Multiple diseases have been associated with aberrant production of chemokines and cytokines, including infectious diseases, chronic inflammation, and autoimmune disorders (1–4).

As part of the large family of cytokines, chemokines act on specific membrane receptors to activate signaling cascades that impact gene expression, thus inducing the production of other factors that contribute to the intricacy of cell-cell interactions. A distinctive characteristic of chemokines is the nature of receptors to which they bind. Unlike cytokines, such as IL-1 that binds to type I transmembrane receptors or TNFα that binds to pre-assembled transmembrane trimers, chemokines act through the activation of G protein-coupled receptors (GPCRs). Binding of chemokines to their cognate seven transmembrane GPCRs triggers structural rearrangements of the receptor that promotes its coupling with heterotrimeric G-proteins, leading to second messenger generation and the activation of intracellular signaling pathways (5–7).

To-date, more than 40 chemokines have been identified in humans, which are in all cases mostly basic, structurally related proteins of 8–14 kDa. Chemokines can be grouped into four classes based on the positioning of their N-terminal cysteine residues: CC, CXC, XC, and CX3C (8–11). The different chemokines play fundamental roles in development, homeostasis, and function of the immune system. Aside from chemotaxis, chemokines are known to have additional functions, including roles in cell proliferation, angiogenesis, and T-cell differentiation. Chemokines are also key players in inflammation, and their levels could be significantly elevated in tissues and plasma of patients with inflammatory conditions. Examples include well-established association between chemokines and diseases such as rheumatoid arthritis, asthma, and psoriasis (12–14).

The causal association between inflammation and cancer has been recognized for decades. Extensive data link chronic inflammatory processes, tissue injury, or infections and the development of cancer. In addition to cancer cells, solid tumors comprise a variety of stromal cells, such as fibroblasts and endothelial cells, as well as inflammatory cells that include lymphocytes, macrophages and neutrophils. Inflammatory cells in the tumor microenvironment functionally interact with cancer cells and other cells within that milieu to impact cancer cell growth and survival, invasion, and metastatic dissemination. In the tumor microenvironment, cancer cells, tumor-associated leukocytes, and stromal cells synergize for the local production of chemokines, among other factors. Tumor-derived chemokines also determine the inflow of leukocytes into the tumor. In the context of tumorigenesis, certain chemokines favor tumor growth and progression, while others boost anti-tumor immunity. The opposite functions of chemokines on tumor development involve attracting cells with pro- or anti-tumoral features (15–19). In this article, we comprehensively review the involvement of the chemokine C-X-C motif ligand 13 (CXCL13) in cancer progression, focusing primarily in novel aspects of CXCL13 biology in solid tumors.

## CXCL13 Chemokine: the Ligand for CXCR5

Interest in chemokines grew tremendously in the early 1980's after the identification of soluble factors responsible for leukocyte and lymphocyte migration and activation. Whereas, early studies identified chemoattractants for monocytes, eosinophils, neutrophils, and T lymphocytes (20–23), B-cell specific chemokines remained enigmatic until the late 1990s, several years after discovery of the receptor for CXCL13, CXCR5 (originally called BLR1) (24).

First identified by comparing gene expression in malignant vs. benign B-cells, BLR1 (Burkitt's Lymphoma Receptor 1) mRNA was detected in Burkitt's lymphoma cell lines and lymphatic tissue but not in undifferentiated B-lymphocytes or hematopoietic cells of myeloid, monocytic, erythroid, or T-lymphocytic origin (24). This finding suggested a role for BLR1 in B-cell development and trafficking into lymphoid tissues, which was later demonstrated in mouse models (25). Because BLR1 mRNA encoded for a protein with a predicted structure containing seven hydrophobic transmembrane segments with a high similarity to the IL-8 receptor, BLR1 was thought to be the first GPCR identified in B-lymphocytes. The physiological importance of this BLR1 receptor was revealed through a gene deletion approach. BLR1-deficient mice failed to develop inguinal lymph nodes and demonstrated severely compromised primary follicle and germinal center formation in the spleen and Peyer's patches (26). This phenotype was explained in experiments assessing the migration pattern of BLR1-deficient B-cells when transferred into wild-type mice, which showed that BLR1^−/−^ B lymphocytes entered T-cell areas of lymphoid tissues but not areas that foster B-cell development that are normally populated by B-cells. Altogether, these studies provided strong evidence that BLR1 was the receptor for a yet-unidentified factor and that its activity facilitated B-cell homing and development in lymphoid tissue.

By searching EST databases for putative CXC chemokines, two simultaneous studies reported the identification of the ligand for BLR1 (27, 28). The novel recombinant chemokine induced B-lymphocyte chemotaxis and it did so only in B-cells expressing BLR1 but not other known chemokine receptors. This was the first identification of a B-cell specific chemokine, and it was termed B cell-Attracting Chemokine 1 (BCA-1). The BCA-1 transcript hybridized most strongly to B-cell-rich follicles in the spleen, germinal centers in Peyer's patches, and lymph node follicles. The BCA-1 protein sequence contained four cysteine residues in a typical CXC chemokine pattern and was located on chromosome 4q21, in close proximity to most other known CXC chemokine genes (29). This chemokine was later renamed CXCL13.

**Figure 1:**
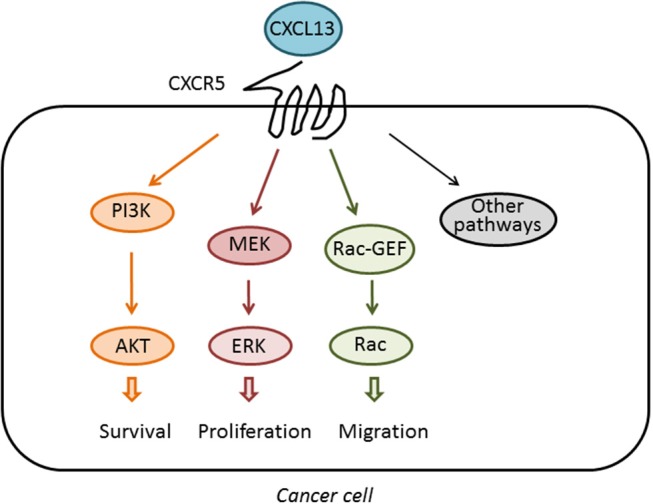
Activation of signaling pathways by CXCL13. The chemokine CXCL13 binds specifically to the GPCR CXCR5. Upon activation, CXCR5 couples to the activation of pathways implicated in cell survival, proliferation, and migration, therefore impacting on the tumorigenic and metastatic activity of cancer cells.

The receptor for BCA-1/CXCL13, later renamed CXCR5, has ~40% amino acid homology to CXCR1, the receptor for IL-8 (30). Building on this sequence homology and the well-characterized structure and function of CXCR1, CXCR5-CXCR1 chimeras were used to dissect the function of the intercellular domains of CXCR5. Similar to CXCR1, CXCR5 activation was found to stimulate intracellular Ca^2+^ influx, ERK/MAPK signaling, and induce cellular chemotaxis (31), an effect that was mediated primarily by the second intracellular domain of CXCR5, called IC2. The CXCR5 IC2 shares 52% amino acid homology with CXCR1, including a DRY motif followed by a conserved YLXIV motif that is common to most chemokine receptors at the junction of the third transmembrane domain and IC2, which facilitates binding to intracellular heterotrimeric G proteins (32). Although the signaling pathways activated downstream of the GPCR CXCR5 have not been fully analyzed, it is known that this receptor couples to the PI3K/Akt, MEK/ERK, and Rac pathways to induce multiple cellular responses, not only in immune cells but also in cancer cells, as it will be discussed in other sections of this review. These pathways are depicted in Figure 1.

## The CXCR5:CXCL13 Axis in the Pathogenesis of Autoimmune Disease

Ectopic germinal center-like lymphoid structures have been recognized within the affected tissues in numerous autoimmune diseases, including myasthenia gravis (33) and rheumatoid arthritis (34), long before the cloning of CXCL13 and CXCR5. After the studies outlined above identified the roles of CXCL13 and CXCR5 in the development of differentiated B-cells and their secondary lymphoid structures, it was natural to hypothesize that aberrant activation of this signaling axis contributes to autoimmune conditions. Abnormal lymphocyte aggregates similar to germinal centers were known to form within the synovium of affected joints in rheumatoid arthritis. Indeed, strong overexpression of CXCL13 mRNA and protein was found in the synovia of patients with rheumatoid arthritis, particularly in the regions of B-cell aggregation (35). Larger-scale analysis of patient synovia demonstrated an extremely high correlation between synovial B-cell rich germinal centers and CXCL13 overexpression, strongly suggesting that CXCL13 signaling contributes to the pathogenesis of these aberrant lymphoid structures (36). Similar contributions of CXCL13 to the formation of ectopic lymphoid structures was later identified in Sjögren's syndrome (37, 38), autoimmune thyroiditis (39), myasthenia gravis (40), systemic lupus erythematosis (SLE) (41), and multiple sclerosis (42). CXCL13 expression has been correlated with disease exacerbation and unfavorable prognosis in multiple sclerosis, Sjögren's (43), myasthenia gravis, and SLE, and CXCL13 has been proposed as a biomarker for diagnosis and progression in these conditions (44).

Whereas, the CXCL13:CXCR5 axis has been best characterized in these autoimmune disorders through the aberrant activity and differentiation of B-cells, numerous other autoimmune conditions appear to be driven by T follicular helper cells T_FH_ cells (45, 46). T_FH_ cells develop from naïve CD4+ T-cells within the interfollicular T cell zone area of lymphoid tissue in response to signals from dendritic cells that cause upregulation of CXCR5 and down-regulation of the T-cell homing marker CCR7 (47). This facilitates relocation of CD4+/CXCR5+/CCR7– T_FH_ cells from T-cell zones to germinal centers within B-cell follicles, where they function primarily to support B-cell expansion and differentiation. Dysregulation of this process in autoimmune disease was first described in SLE (48), wherein an aberrant overabundance of T_FH_ cells can cause spontaneous germinal center formation and autoimmunity (49). Indeed, circulating CXCR5+/CD4+ T-cells that resemble T_FH_ cells have been identified in SLE patients, where they facilitate pathologic B cell differentiation and correlate with disease progression. Pathogenic roles for T_FH_ cells have also been reported for many other autoimmune entities such as Sjögren's syndrome (50), primary biliary cholangitis (51), vitiligo (52), ankylosing spondylitis (53), and pemphigus vulgaris (54). Overall, dysregulation of the CXCL13:CXCR5 axis affecting both B- and T_FH_ cell function is major player in autoimmune disorders, and potentially serves as a biomarker for disease progression and therapeutic response.

## CXCL13 and CXCR5 in Infectious Disease

Based on the crucial roles the CXCL13:CXCR5 axis plays in both physiologic and pathologic immunity, it is not surprising that this axis has been implicated in the pathogenesis of a number of infectious diseases. Before CXCL13 had been cloned, CXCR5 was identified as a co-receptor for HIV-2 that renders T_HF_ cells susceptible to viral infection. Expression of CXCR5 and CXCL13 was shown to be dysregulated in HIV infection, such that the number of CXCR5+ B cells decreases with progression of HIV infection, together with an increase in plasma levels of CXCL13 (55). Later studies confirmed elevations of serum CXCL13 during chronic HIV infection and showed that CXCL13 levels correlated both with disease progression and viral load. CXCL13 levels decrease after highly active antiretroviral treatment (HAART) (56).

Mechanistically, a loss of CXCR5+ B-cells in HIV viremia was shown to drive expansion of CXCR5+ T_FH_ cells within lymph nodes. These T_FH_ cells secrete cytokines, such as IL-21, that induce plasma cell differentiation and immunoglobulin secretion, namely IgG1, explaining the B cell dysfunction and hypergammaglobulinemia observed in chronic HIV infection (57). Later studies confirmed expansion of CXCR5+ T_HF_ cells within lymph nodes during HIV infection and demonstrated that T_FH_ cells serve as the major *in vivo* reservoir for HIV infection, replication, and production (58). Whereas, T_FH_ cells within lymphoid tissues are expanded, the frequency and functionality of peripheral CXCR5^+^ T_FH_ cells has been shown to decline during chronic HIV-1 infection. A subset of CXCR5+ T_HF_ cells that also express programmed death-1 (PD-1) have been shown to facilitate the development of a strong B-cell response in early HIV infection (59), and preservation of peripheral CXCR5+ T_FH_ cells correlate with long-term control of infection. Finally, by demonstrating temporal correlation of CXCL13 plasma levels with progression of HIV infection, CXCL13 was proposed as a biomarker of systemic immune activation during HIV infection that may serve to predict AIDS-defining events (60).

It is clear that the CXCL13:CXCR5 axis is intimately involved in the initial and chronic phases of HIV infection, and, considering the central role this axis plays in humoral immunity, it is not surprising that CXCL13 has been implicated in the pathogenesis of several other infectious diseases. Of these, perhaps the best studied are the stages of Lyme disease and syphilis affecting the central nervous system (CNS) (Lyme neuroborreliosis and neurosyphilis). CXCL13 is overexpressed within the muscles of monkeys chronically infected with *Borrelia burgdorferi* (the etiological agent of Lyme disease), and CXCL13 was later shown to contribute to the formation of ectopic germinal centers within the central nervous system. Interestingly, whereas infection with *B. burgdorferi* appears to have no impact on plasma CXCL13 levels, once the bacteria establishes CNS infection, it leads to constitutively elevated CXCL13 levels in cerebrospinal fluid (CSF), which could be often more than several 100-fold greater than in the plasma (61). CXCL13 appears to recruit B-cells within the CNS and facilitate their differentiation to plasma cells that produce a burgdorferi-targeted humoral response. Indeed, CSF CXCL13 level has been proposed as a diagnostic biomarker for neuroborreliosis, and a recent meta-review of 18 studies calculated a pooled sensitivity and specificity of 89 and 96%, respectively, for CNS CXCL13 as a biomarker of disease (62).

Similar to neuroborreliosis, CXCL13 has been implicated in the pathogenesis of neurosyphilis, a serious complication of untreated syphilis. Neurosyphilis is typically a late manifestation of prolonged infection but can also occur in early disease, and it generally manifests as chronic meningitis, stroke-like symptoms, or neurological symptoms (dementia, tabes dorsalis, and paresis). Notably, CSF levels of CXCL13 were found to be 100-fold higher in patients infected with *Treponema pallidum* (the etiological agent of syphilis) than in uninfected individuals, although approximately four times lower than individuals with neuroborreliosis. Mechanistically, enrichment and activation of B-cells have been observed within the CNS in neurosyphilis, as well as ectopic germinal centers, suggesting that CNS *T. pallidum* infection leads to CXCL13 overexpression and a positive feedback loop that recruits and activates a strong humoral response within the CNS that contributes to destruction of neurological tissue (63). Similar to neuroborreliosis, CSF levels of CXCL13 have been proposed as a biomarker for neurosyphilis with a sensitivity and specificity of 85 and 89%, respectively (64), with the highest diagnostic value being in HIV-infected patients (65).

## CXCL13 in Lymphoproliferative Diseases and Lymphoma

As outlined in the previous section, CXCL13 is strongly expressed by dendritic cells in the follicles within the spleen, lymph nodes, and Peyer's patches, where it binds to CXCR5 on mature B cells and T_HF_ cells to facilitate the development of these B cell-rich structures and B-cell differentiation. Imbalances in the CXCL13:CXCR5 axis may contribute to pathologies involving B-cells and T_HF_ cells. Early studies revealed that CXCL13 and CXCR5 are highly expressed in primary and secondary follicles within gastric lymphomas (66). Malignant cells in follicular lymphoma, which mimics the architectural and cellular structures of normal secondary lymphoid follicles in ectopic neoplastic foci, were shown to express CXCR5, secrete CXCL13, and migrate in response to CXCL13, suggesting that CXCL13 recruits malignant B-cells to ectopic germinal centers and contributes to their development (67). Shortly thereafter, overexpression of CXCL13 or CXCR5 was demonstrated in primary central nervous system B-cell lymphoma (68), cutaneous B and T-cell lymphoproliferative disorders, intraocular lymphomas, and chronic lymphocytic leukemia (69).

Just as CXCL13 has demonstrated potential clinical utility as a biomarker in infectious diseases involving the CNS, this chemokine has been also proposed as a marker of certain lymphomas. One of the strongest correlations has been demonstrated for angioimmunoblastic T-cell lymphoma (AITL), an aggressive nodal T cell lymphoma that accounts for approximately 1.5% of all non-Hodgkin lymphoma and 20% of peripheral T cell lymphomas. Because AITL is derived from T_FH_ cells, CXCL13 was first suggested as a biomarker for this disease (70), and the 2016 WHO classification of lymphomas included CXCL13 in the diagnostic criteria for AITL (71). Elevated CXCL13 levels in the CSF have been demonstrated for CNS lymphomas (72), and when elevated together with IL-10 in the CSF, CXCL13 has a >99% specificity for primary and secondary lymphomas, leading to similar proposals that it serves as a biomarker for non-Hodgkin lymphoma involving the CNS (73).

**Figure 2:**
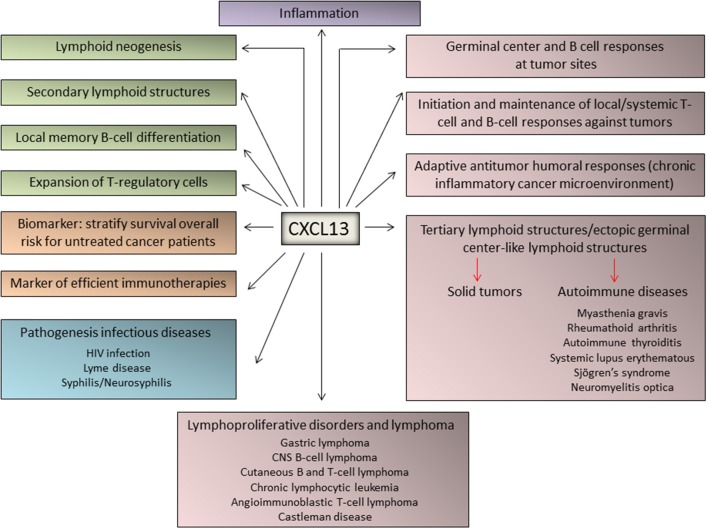
Major functions of CXCL13 in immune responses, inflammation, and lymphoproliferative diseases. The chemokine CXCL13 has been identified as a major regulator of immune responses and plays key roles in the pathophysiology of inflammatory, infectious, and lymphoproliferative diseases. In addition, emerging evidence identified CXCL13 as a biomarker for cancer progression and response to therapy.

Very recently, CXCL13 was identified as the most up-regulated cytokine in plasma from patients with idiopathic multicentric Castleman Disease, a poorly understood syndrome involving a “cytokine storm” driving polyclonal lymphoproliferation. Elevated CXCL13 plasma levels has been demonstrated in lymph nodes from these patients, likely in follicular dendritic cells and T_FH_ cells (74). A schematic representation of the major physiological and pathological functions of CXCL13 is depicted in Figure 2.

## CXCL13:CXCR5 Axis in Solid Tumors

### CXCL13:CXCR5 Involvement in Tertiary Lymphoid Structure Formation in Tumors

**Figure 3:**
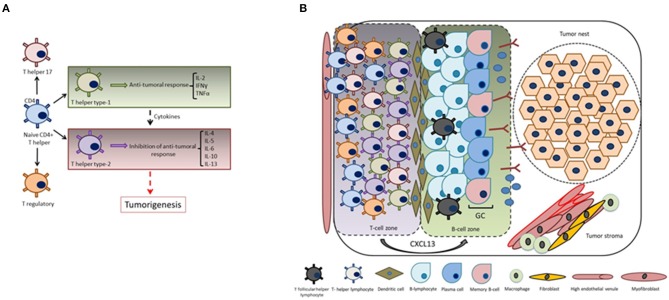
CXCL13 and the formation of tertiary lymphoid structures. **(A)** Cytokines play key roles in the immunomodulation of both T helper type-1 (cellular immunity) or T helper type-2 responses (humoral immunity). The shift from an immunological pattern with a T helper type-1 orientation to a T helper type-2 pattern mediated by cytokines is a key event in tumorigenesis. **(B)** CXCL13 produced by T-cells plays a fundamental role in the formation of tumor-associated tertiary lymphoid structures and germinal center orchestration: tumor infiltrating T-cells are recruited into the tumor site by transendothelial migration via high endothelial venules mediated by chemokine/chemokine receptor interactions. Intra-tumoral infiltration of T_FH_ cells is a key step in the formation of tertiary lymphoid structures, thus generating and promoting B-cell responses in the germinal center (i.e., local memory B cell differentiation, as well as with the expansion of a subpopulation of T regulatory cells).

Naive CD4+ T helper precursor cells can differentiate into a variety of different T helper subsets, including T helper type-1 (exerting antitumoral responses), T helper type-2 (displaying inhibition of antitumoral responses), T helper 17 and regulatory T-cells. Noteworthy, T helper type 1-cells produce a particular set of cytokines such as IL-2, IFNγ, and TNFα, while T helper type-2 cells produce IL-4, IL-5, IL-6, IL-10, and IL-13. It has been reported that a shift from an immunological pattern with a T helper type-1 orientation to a T helper type-2 pattern mediated by cytokines is a key event in carcinogenesis (75, 76) (Figure 3A).

Tertiary lymphoid structures present in the tumor microenvironment of solid tumors are basically characterized by mature dendritic cells in a T-cell zone adjacent to B-cell follicle including a germinal center representing sites of lymphoid neogenesis (77–79) (Figure 3B). These tertiary lymphoid structures are essential sites for the initiation and/or maintenance of the local and systemic T- and B-cell responses against tumors and associate with a favorable clinical outcome for cancer patients. Hence, they can be considered novel biomarkers to stratify the overall survival risk of untreated cancer patients and as markers of efficient immunotherapies (80). Going deeper into the analysis of tertiary lymphoid structure neogenesis, several studies revealed an important participation of the CXCL13:CXCR5 axis in this process. For instance, the presence of functional tertiary lymphoid structures has been associated with long-term survival in lung cancer patients, and signaling by CXCL13, among other chemoattractants, has been shown to mediate T-cell recruitment to tertiary lymphoid structures (81). Regarding the orchestration of the T-cell migration mechanisms, it was demonstrated that T-cells express CXCR5. A new gateway mechanism proposed for T-cell migration into the tumor involves their recruitment via high endothelial venules mediated by chemokine/chemokine receptor interactions, thus reinforcing the concept that recruitment of tumor specific T-cells to intratumoral tertiary lymphoid structures is mediated by the CXCL13:CXCR5 axis (82).

**Figure 4:**
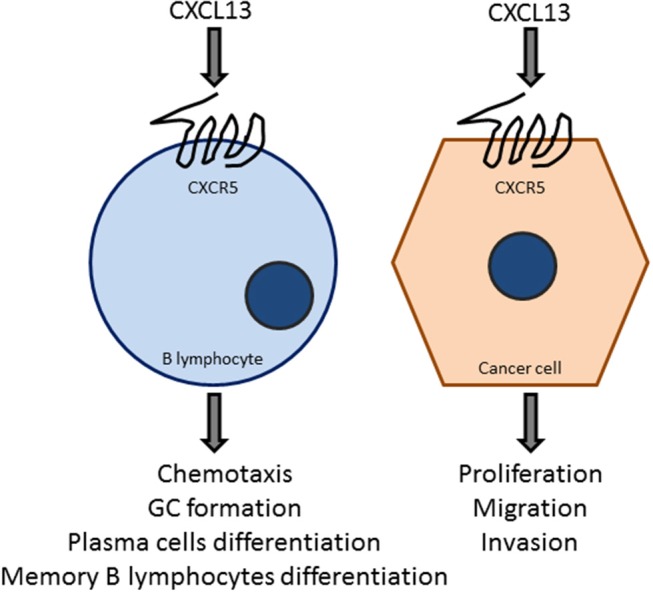
The CXCL13:CXCR5 axis is a key element in B-cell and tumor cell responses. The chemokine CXCL13, acting on the GPCR CXCR5, promotes chemotaxis, germinal center formation, and the differentiation to plasma cells and B-memory lymphocytes. CXCL13 also target cancer cells to promote proliferation, migration, and invasion. These effects vary depending on the cancer type, which may express different levels of CXCR5.

Infiltration of TFH cells (83) is important for tertiary lymphoid structure formation and to generate germinal center B cell responses at the tumor site by the production of CXCL13. These tumor-infiltrating CXCL13-producing T_FH_ cells were linked with promoting local memory B cell differentiation, as well as with the expansion of a subpopulation of T regulatory cells. Moreover, these cells were also related with the *de novo* activation of adaptive antitumor humoral responses in the chronic inflammatory breast cancer microenvironment. CXCL13:CXCR5 axis is involved in many biological responses in immune cells as well as in cancer cells (Figure 4), as described in subsequent sections.

### Role of CXCL13:CXCR5 Axis in Lung Cancer

Lung cancer is the leading cause of cancer-related deaths worldwide. The immune system exerts a central role in the lung cancer biology given, at least in part, by its pro-inflammatory milieu. While several studies provided solid evidence supporting the starring role of the CXCL13:CXCR5 axis in the promotion of lung tumor progression, studies focused on CXCL13 produced by immune cells also highlight its high predictive potential for response to immunotherapy.

CXCL13 was initially identified as part of an invasive network module in lung adenocarcinomas (84–86). A comparative analysis of an inflammatory signature in non-small cell lung cancer (NSCLC) and chronic obstructive pulmonary disease (COPD) patients revealed that CXCL13 was included among the chemokines elevated in NSCLC patients (86, 87). An inflammation score based on CXCL13 and three additional markers (CRP, MDC/CCL22, and IL-1RA) provided good separation in 10 year cumulative risks of lung cancer. CXCL13 was also identified as a predictive factor for risk of early stage lung adenocarcinoma (88). A specific gene expression signature associated with T-cell presence in tertiary lymphoid structures was identified in human lung cancer, which includes CXCL13 and other chemokines (89).

One interesting recent study focused on the prognostic relevance of tertiary lymphoid structures in lung squamous cell carcinoma patients treated with corticosteroid together with chemotherapy (90). This study identified a perivascular CXCL13 positive niche that supports tertiary lymphoid structure development, which is associated with improved patient survival. Steroid treatment impaired the formation of these lymphoid structures when compared with those untreated patients, who showed high tertiary lymphoid structures intratumoral density, as well as the expression of adaptive-immune response genes. This led to the hypothesis that corticosteroid treatment as neoadjuvant therapy abrogates germinal center formation with the subsequent loss of the tertiary lymphoid structures (90). Recent evidence also showed that CD8+ lymphocyte populations with high PD-1 expression from NSCLC patients express and secrete very high levels of CXCL13. Secretion of CXCL13 by the tumor infiltrating lymphocytes expressing high PD-1 levels serves to attract other immune cell subsets to the tumor microenvironment, including T_FH_ cells and B-cells, and, strikingly, it strongly predicts response to anti-PD-1 therapy that correlates with increased overall survival and durable responses. Thus, CXCL13 and the immune cells that produced this chemokine in the NSCLC tumor microenvironment may represent novel biomarkers for response to targeted PD1/PD-L1 therapy (91).

Compelling data for the involvement of CXCL13 in lung cancer development was provided by Wang et al. (92), who studied the relationship between lung cancer development and environmental pollution in Xuanwei, a city in the Yunnan Province with one of the highest lung cancer incidences in China. This has been attributed to smoky coal combustion-generated polycyclic aromatic hydrocarbons (PAHs) pollution. A screening analysis of abnormally high inflammatory factors in NSCLC patients from this region revealed that 90% of the tumor tissues have elevated CXCL13 levels. CXCR5 could be also detected in human lung tumors, however its levels were not significantly different between cancer and normal tissue. The causal link between carcinogens and CXCL13 production was confirmed using a normal human lung epithelial exposed to the PAH benzo(a)pyrene (Ba[a]P), which caused elevated transcriptional activity of the *CXCL13* gene via the aryl hydrocarbon receptor (AhR), a ligand-activated transcription factor that binds to a xenobiotic-responsive element (XRE) in this gene located 1.7 kb downstream from the transcription start site. Lung tumors from mice treated with Ba[a]P display elevated CXCL13 mRNA and protein levels. The relevance of CXCL13 in Ba[a]P-induced lung cancer was further confirmed using CXCL13 and CXCR5 knockout mice, which have impaired tumor formation in response to the carcinogen. CXCL13 within the tumors was produced by CD68+ macrophages. CXCL13 markedly increase the production of the macrophage-secreted cytokine SPP1 (secreted phosphoprotein-1 or osteopontin). The potential involvement of SPP1 was confirmed by silencing its expression, which led to impaired migration of lung cancer cells in co-culture models. CXCL13 and SPP1 serum concentrations were elevated in B[a]P-treated mice, and tumors show elevated SPP1 staining. Notably, the CXCL13-CXCR5-SPP1 signal induced an EMT phenotype in lung tumors from B[a]P-treated mice, which was characterized by E-cadherin down-regulation and up-regulation of mesenchymal markers N-cadherin, vimentin, Slug and Snail. SPP1 overexpression was also linked to increased levels of nuclear β-catenin.

Another study in NSCLC patients revealed significantly higher expression of CXCR5 in carcinomas relative to non-neoplastic lung tissue (93). Further, nuclear and membrane CXCR5 intensities were higher in NSCLC relative to non-neoplastic tissues. The relevance of the nuclear CXCR5 staining is unknown. Interestingly, this study also revealed higher levels of CXCL13 in serum of NSCLC patients compared to healthy controls. The functional significance of the high CXCR5 expression was analyzed using migration assays, which revealed a pro-migratory phenotype in NSCLC cells subject to CXCL13 stimulation. These results suggest that CXCL13 in the tumor microenvironment may act upon the cancer cell, possibly contributing to tumorigenic and metastatic phenotypes. It would be of great interest to determine whether lung cancer cells respond to CXCL13 by activating signaling pathways related to proliferation and survival, as this may reveal important direct effects of the chemokine resulting from paracrine or autocrine mechanisms.

### Relationship Between the CXCL13:CXCR5 Axis and Breast Cancer Progression

Breast cancer exhibits one of the strongest relationships between CXCL13:CXCR5 axis and tumor progression. Using a microarray analysis, a study found significant CXCL13 overexpression in breast cancer specimens (94). Moreover, CXCL13 was identified as the most strongly overexpressed chemokine in breast cancer tissue compared with normal breast tissue. A positive correlation was found between the expressions of CXCL13 and CXCR5 in breast tumors. Analysis in breast cancer cell lines also revealed high levels of CXCL13 and expression of CXCR5. Strikingly, both ligand and receptor could be only detected intracellularly in cell lines growing in culture, with no measurable levels of the CXCL13 in the culture medium (by ELISA) or plasma membrane CXCR5 (by flow cytometry). Nonetheless, elevated serum concentrations of CXCL13 could be detected in patients with metastatic disease, possibly reflecting differences between 2-D cultures and tumors. Biswas et al. reported high co-expression of CXCL13 and CXCR5 within primary breast tumors, as determined by immunohistochemistry (95). Chemokine and receptor up-regulation are driven by different mechanisms involving transcriptional control via RelA and Nrf2 in the case of the *CXCL13* gene, and epigenetic regulation (lack of CpG island methylation) in the case of the *CXCR5* promoter (95). Along the same line, a study revealed high CXCL13 expression in primary tumors from Chinese young breast cancer patients (≤45 yo) but not in those from older women (≥65 yo). Moreover, CXCL13 expression was associated with grade 2/3, lymph node positive and ER negative status (96). Despite the differential CXCL13 expression found in breast cancer, a study failed to show elevated CXCL13 plasma levels in breast cancers patients relative to healthy controls (97). CXCL13 was also included in a set of 14 prognostic gene predictors of longer metastasis-free survival in early stage hormone receptor-negative and triple-negative breast cancer (TNBC) (98).

The analysis of a T-cell signature in breast cancer that includes CXCL13 revealed additional levels of complexity. In univariate analysis, mRNA expression of CXCL13 and other T-cell markers associate with longer metastasis free-survival, with a stronger prognostic effect in HER2 positive breast cancer (99). In a recent study, a four-gene signature that includes CXCL13 and predicts the extent of lymphocytic infiltration after neoadjuvant therapy in TNBC has been developed. This signature associates with good outcome, adding novel prognostic information for this aggressive breast cancer subtype (100). Likewise, the recent association between high CXCL13 and distant disease-free survival in early-stage breast cancer patients provided evidence that humoral immunity influences the survival outcomes in these patients, particularly those with TNBC (101).

In addition to the immune-related effects of CXCL13 in breast cancer, this chemokine exerts direct effects on breast cancer cells. In an early study using CXCR5-expressing breast cancer cells, CXCL13 induced changes in the expression of markers consistent with epithelial-to-mesenchymal transition (EMT). Indeed, CXCL13 up-regulates mesenchymal markers vimentin, Snail, Slug, N-cadherin, MMP9 and RANKL while down-regulating E-cadherin expression. In addition, CXCL13-treated breast cancer cells acquire a more elongated shape and a highly migratory phenotype characteristic of mesenchymal cells. From a signaling perspective, these effects are sensitive to p110α PI3K and Src inhibition (95). Interestingly, a recent study showed that an anti-CXCL13 antibody reduces MDA-MB-231 breast cancer cell viability by promoting apoptosis. CXCL13 inhibition reduced active ERK and cyclin D1 levels, and enhanced caspase-9 cleavage (47). Extra backing proof of the concept that CXCL13 plays a pivotal role in breast cancer growth and lymph node metastasis was gained in tumorigenesis experiments using 4T1 breast cancer cells. Treatment of mice with an anti-CXCL13 antibody impaired 4T1 tumor growth and ERK activation, thus affording a theoretical frame for clinical trials targeting CXCL13 (102).

An inverse correlation between the expression of CXCR5 and the p53 tumor suppressor was reported in the MCF-7 human breast cancer cell line. Silencing of p53 in these cells not only up-regulates CXCR5, but also potentiates CXCL13-mediated chemotaxis. Therefore, CXCR5 up-regulation may contribute to the anomalous phenotypes of p53-deficient cells. A CXCR5 promoter analysis revealed that p53 acts indirectly by repressing the activity of NF-κB transcription factors (103). Similar mechanisms for CXCR5 gene regulation may operate upon loss of related tumor suppressors p63 and p73 (104). These studies suggest a potential relationship between genotoxic stress and CXCR5 responses that could have significant prognostic and therapeutic implications in the context of chemotherapy.

### CXCL13:CXCR5 Axis and the Progression of Gastrointestinal Tract Tumors

Colorectal carcinoma is one of the most common malignancies worldwide. Extensive analysis of circulatory inflammatory factors at the time of colorectal cancer surgery, including cytokines, chemokines and interleukins, revealed CXCL13 as one of the factors associated with increased mortality (105). In agreement with this information, immunohistochemical analysis of advanced colorectal cancer specimens revealed significant elevation of CXCL13 and CXCR5 in tumors relative to normal tissue, which correlated with lymph node metastasis and neural invasion. Furthermore, patients with positive CXCR5 and CXCL13 expression have poor prognosis, both in terms of 5 year overall survival and 5 year relapse-free survival. Interestingly, CXCR5 staining localized primarily in the epithelial cells, whereas CXCL13 staining was mainly found in the mesenchymal cells (106). These results suggest a role of CXCR5 and CXCL13 as prognostic markers for colorectal carcinoma progression.

From a functional standpoint, CXCL13 induces proliferation and migration in CXCR5-expressing colon cancer cells (107). Interestingly, CXCR5 expression in mouse CT26 colon carcinoma was low *in vitro*, up-regulated *in vivo*, and rapidly lost when cells were explanted *in vitro*. In the liver, after intrasplenic injection, these CXCR5 transfectants initially grew faster than controls; however, the growth rate of control tumors accelerated later to become similar to the transfectants, likely due to CXCR5 up-regulation. These results suggest that expression of CXCR5 in tumor cells promotes their growth in the liver and, at least for CT26 cells, the receptor is required for outgrowth to large liver tumors. CXCL13 also promotes growth, migration, and invasion in human SW620 colon carcinoma cells. From a signaling standpoint, CXCL13 activates, and the migratory/invasive phenotype is sensitive to PI3K inhibition (108). This has significant clinical implications since abnormal PI3K signaling is a hallmark of colon cancer (109). Studies using the Nirp12 KO mouse model revealed that elevated CXCL13 expression occurs as a consequence of high non-canonical NF-κB activation (85). As in other cancers, CXCL13 and its receptor also play roles within the immune landscape of colon cancers (110).

In gastric cancer, CXCL13 expression is predictive of shorter overall survival. Within the T2–T4 stage patient group, low CXCL13 expression is associated with longer survival, particularly in patients who received adjuvant chemotherapy. High CXCL13 levels in gastric tumors are associated with larger tumor diameters (111). Notably, a transcriptome analysis of biopsies from gastric cancer patients revealed significant up-regulation of CXCL13, which is mainly expressed in isolated lymphoid follicles and small lymphoid aggregates (112). In gastric biopsies from *H. Pylori* infected patients, who have increased risk of developing gastric carcinoma, there is also significant CXCL13 overexpression (113). This finding fits with an early analysis in *H. Pylori* gastritis showing that CXCL13 expression was mainly confined to lymphocyte aggregates as well as to primary and secondary follicles. Analysis of CXCL13 expression in mucosa-associated lymphoid tissue (MALT) lymphomas shows that the transformed blasts seem to be the major source of the chemokine (66). Consistent with observations in other cancers, serum levels of CXCL13 were higher in patients with gastric cancer compared to healthy individuals, and this correlates with a high number of circulating T_FH_ cells. Notably, CXCL13 concentrations were higher in patients with lymph node metastasis and high-grade disease (114).

CXCL13 also seems to play a prominent role in the development of pancreatic adenocarcinoma, one of the most aggressive and incurables forms of cancer. Human pancreatic carcinoma cell lines express CXCR5, and this GPCR can be detected in a significant proportion of human pancreatic tumors (107). Several pancreatic ductal adenocarcinoma cell lines display significant elevation of non-canonical NF-κB target genes, including CXCL13. Constitutive activation of the non-canonical NF-κB pathway requires stabilization of the kinase NIK and IKKα-dependent processing of NF-κB2/p100 to p52, which via heterodimerization with RelB heterodimers regulates the expression of genes encoding lymphoid-specific chemokines and cytokines (115). Constitutive activation of the non-canonical NF-κB pathway as observed in pancreatic cancer models may contribute to a positive pro-migratory autocrine loop via CXCR5 activation, as observed in prostate cancer models (see below). More recently, the Simon laboratory identified a pathway in KRas-driven pancreatic tumors that involves CXCL13 and the master regulator of the hypoxic transcriptional response HIF1α. Deletion of the *Hif1a* gene in mice surprisingly accelerates pancreatic carcinogenesis, which was accompanied by increased infiltration of B lymphocytes. Interestingly, one of the chemokines up-regulated by HIF1α was CXCL13. Depletion of B cells by administration of a CD20-specific monoclonal antibody reduced the number of lesions (116). Taking into consideration that CXCL13 staining can be detected in the stroma surrounding human and mouse PanIN lesions, most likely in the stromal fibroblast population, and that CXCL13 neutralization with a blocking antibody reduces pancreatic B cell infiltration and the growth KRas-driven tumors (117), it is logical to speculate that CXCL13 has a significant contribution to pancreatic tumorigenesis.

Hepatocellular carcinoma (HCC), the primary malignant tumor of the liver, has been highly associated with chronic inflammation related to alcohol intake and viral hepatitis. Many cytokines and chemokines have been linked to chronic liver disease and HCC, including CXCL13. Elevated CXCL13 and CXCR5 expression has been reported in human HCC, with a higher percentage of CXCR5+ or CXCL13+ cells in poorly differentiated tumors compared with well-differentiated tumors (118). Moreover, studies documented elevated CXCL13 serum levels in HCC patients, which correlate with tumor size, metastatic disease, advanced stages, and Alanine Transaminase/Aspartate Aminotransferase serum levels (118, 119). Nonetheless, in hepatitis B-related HCC, elevated serum CXCL13 did not correlate with overall survival and rather correlated with recurrence-free survival (118). Analysis of signaling networks revealed a mutual positive interaction between CXCL13 and the Wnt/β-catenin pathway in promoting liver cancer (119). These studies emphasize the potential roles of the CXCL13:CXCR5 axis as a biomarker in HCC and its potential prognostic relevance.

### CXCL13:CXCR5 and the Development of Prostate Cancer

Prostate cancer is the most commonly diagnosed malignancy and the second leading cause of cancer death in men. Metastasis, particularly to the bone, occurs in most patients when disease becomes androgen-independent (“castration-resistant prostate cancer” or CRPC). Within the last decade, chemokines emerged as key players in prostate cancer progression. In this context, CXCL13 and CXCR5 appear to be highly relevant in prostate cancer cell proliferation, migration, and invasion, ultimately impacting disease progression and metastatic dissemination.

Early studies showed that CXCR5 is expressed in primary prostate cancer tissues at higher levels than normal tissue. In normal tissue and benign prostate hyperplasia samples, CXCR5 displays a predominant membrane and/or cytoplasmic distribution while in advanced prostate cancers it shows high nuclear expression (120). CXCL13 is elevated in serum of prostate cancer patients and was found to be a better predictor of prostate cancer than prostate-specific antigen (PSA). Also, CXCL13 is highly expressed in human bone marrow endothelial cells and osteoblasts, but not in osteoclasts, in response to IL-6 treatment. Furthermore, CXCL13 produced by bone marrow endothelial cells in response to IL-6 was able to induce prostate cancer cell invasion in a CXCR5-dependent manner (120).

Signaling studies revealed that DOCK2 (Dedicator of cytokinesis 2), ERK1/2, JNK and Akt signals mediate CXCL13 invasive and proliferative responses in prostate cancer cells. CXCL13 promotes proliferation in androgen-responsive LNCaP cells in a JNK-dependent, DOCK2-independent manner, whereas this effect is dependent on DOCK2 in androgen-independent PC3 cells. CXCL13-mediated invasion in prostate cancer cells depends on PI3K/Akt, Src, ERK, and FAK, but it is independent of DOCK2 (121, 122). Interestingly, androgen stimulates CXCL13 production in prostate cancer cells, and *CXCL13* was found to be an androgen-responsive gene that contains a canonical androgen-responsive element in its promoter. From a functional standpoint, CXCL13 plays an important role in androgen receptor-induced cellular migration and invasion in LNCaP cells (123).

**Figure 5:**
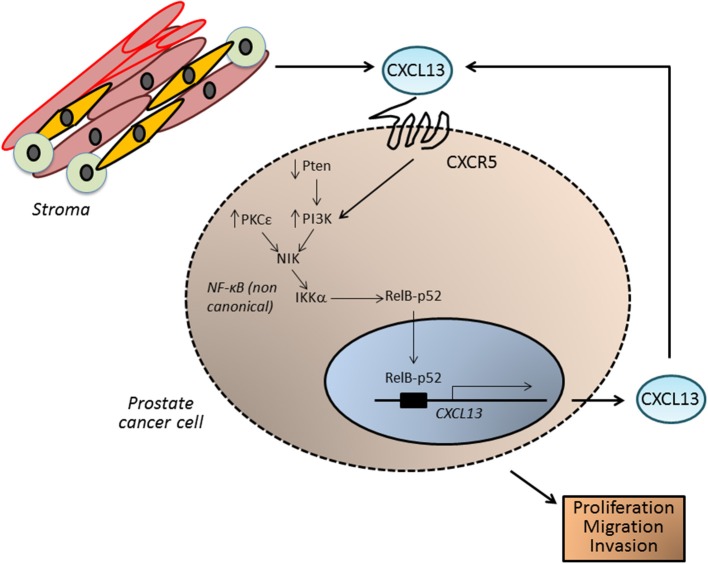
CXCL13 in prostate cancer. In prostate tumors, CXCL13 can be produced both by cancer cells as well as by cells in the tumor microenvironment, such as myofibroblasts. In prostate cancer cells, up-regulation of the kinase PKCε and loss of the tumor suppressor Pten (which leads to elevated PI3K activity) lead to the activation of the non-canonical NF-κB pathway, and transcriptionally activate the *CXCL13* gene. CXCL13 produced in this autocrine manner, together with CXCL13 generated by stromal cells, may significantly impact on the tumorigenic and metastatic phenotypes of androgen-independent prostate cancer cells.

Our laboratory has recently implicated the oncogenic kinase PKCε in prostate cancer progression (124–127). This kinase is overexpressed in prostate cancer and cooperates with loss of the tumor suppressor Pten, a common alteration in prostate cancer, for the development of prostatic adenocarcinoma (128, 129). Gene profiling of PKCε-overexpressing/Pten-deleted prostate epithelial cells revealed CXCL13 as a top deregulated gene. PKCε overexpression and Pten loss (which leads to PI3K activation) up-regulates CXCL13 production and release, contributing to CXCR5 signal amplification, and ultimately resulting in a cell autonomous pro-migratory and tumorigenic autocrine loop. Indeed, silencing CXCR5 from prostate cancer cells reduces their proliferative, tumorigenic and motile capacities. CXCL13 up-regulation in prostate cancer cells is driven by the non-canonical NF-κB pathway. A responsive element for the non-canonical NF-κB pathway has been identified in the *CXCL13* gene promoter, and *CXCL13* promoter transcriptional activity is sensitive to pharmacological inhibition of PKC, PI3K, and NF-κB. Furthermore, silencing IKKα and NIK, key elements in the non-canonical NF-κB pathway, down-regulates CXCL13 mRNA levels and *CXCL13* gene transcriptional activity (128) (Figure 5).

Using a Myc-CaP mouse model, Karin and coworkers studied the involvement of CXCL13 in CRPC. After castration, there was elevated expression of CXCL13 in myofibroblasts within the tumor remnants. Immunoablation of FAP (fibroblast activation protein)-expressing cells led to the disappearance of myofibroblasts that express CXCL13 in the tumor stroma of androgen-deprived Myc-CaP tumors, reduced the infiltration of T cells, B cells and dendritic cells into the tumor remnants, and retarded the evolution of CRPC. Mechanistically, FAP ablation prevented IKKα nuclear translocation in cancer cells. Blockade of TGF-β signaling abrogated not only B and T cell infiltration but also the induction of CXCL13-expressing myofibroblasts. Interestingly, cultured fibroblasts isolated from Myc-CaP tumors from non-castrated mice responded to hypoxic conditions by converting into myofibroblasts that produced CXCL13, an effect that is sensitive to phosphodiesterase 5 (PDE5) inhibition with sildenafil. Sildenafil significantly delayed CRPC in castrated Myc-CaP tumor-bearing mice. Myofibroblast activation, immune infiltration, and induction of TGF-β and CXCL13 could be also observed upon castration of TRAMP mice, a model that develops metastatic tumors with neuroendocrine differentiation. This study also identified higher expression of CXCL13 and nuclear HIF-1α in malignant prostate tissue compared with normal tissue or benign prostatic hyperplasia. Furthermore, B cells in malignant tissues were located next to CXCL13-expressing cells. These findings suggest that B lymphocytes recruited into androgen-deprived tumors by CXCL13 play an important role in malignant progression and metastatic dissemination of prostate cancer (130).

Altogether, evidence indicates that a complex network of cellular interactions involving CXCL13 and CXCR5 integrate to promote prostate cancer cell autonomous and non-autonomous pathways. These findings merit further translation into preclinical and clinical arenas, since targeting the CXCL13:CXCR5 axis may be a promising approach for the treatment of CRPC. Recent discoveries linking up-regulation of the CXCL13:CXCR5 axis to the dissemination of prostate cancer stem-like cells to lymph nodes and bone marrow (131) further support this concept.

### Involvement of the CXCL13:CRXR5 Axis in Other Solid Tumors

A very recent study showed significant CXCL13 up-regulation in clear renal cell carcinoma (ccRCC) that correlates with advanced disease stage and poor prognosis, together with elevated CXCL13 serum levels in ccRCC patients. Receiver Operating Characteristic (ROC) curves showed that tissue and serum CXCL13 expression may represent a useful diagnostic biomarker for ccRCC. Notably, patients in the high CXCL13/high CXCR5 expression group have a worse prognosis. CXCL13 promotes the proliferation and migration of ccRCC cells and activates the PI3K/Akt/mTOR signaling pathway. Thus, the CXCL13:CXCR5 axis plays a significant role in ccRCC and could be a valuable therapeutic target and prognostic biomarker (132).

The CXCL13:CXCR5 axis has also been implicated in the initiation and progression of other solid tumors, such as ovarian cancer, melanoma, oral squamous cell carcinoma, osteosarcoma, thyroid cancer, and neuroblastoma (133–143). One interesting study showed that specific chemokine signatures may contribute to overall survival in wild-type and mutant p53 ovarian cancers, and CXCL13 was specifically associated with better overall survival (137). Also, ascites in obese mice have higher levels of macrophages and chemokines including CXCL13, suggesting that obesity may accelerate the peritoneal dissemination of ovarian cancer through higher production of pro-inflammatory chemokines and macrophages recruitment (138). CXCL13 has been also implicated in oral squamous cell carcinoma tumor progression and osteolysis. The tumor necrosis factor family member RANKL (Receptor Activator of NF-κB ligand) plays an important role in cancer invasion of bone/osteolysis. High CXCL13 expression levels have been reported in primary human oral squamous cell carcinoma tumors. c-Myc activation through the CXCL13:CXCR5 signaling axis stimulates RANKL expression in stromal/preosteoblast cells, therefore implicating CXCL13 as a potential therapeutic target to prevent oral squamous cell carcinoma invasion of bone/osteolysis (140).

Of note, immune dysregulation plays a key role in the development of osteosarcoma. Peripheral blood CD4+CXCR5+ T-cells induce B-cell activation and produce a number of cytokines that play critical roles in tumorigenesis. Patients with high tumor grade have an elevated percentage of CD4+CXCR5+ T-cells compared to those with low tumor grade. Moreover, Th1 and Th17 subtypes contribute to the upregulation of peripheral CD4+CXCR5+ T-cells in patients with metastasis or high tumor grade. These results argue for the involvement of peripheral CD4+CXCR5+ T-cells and the CXCL13 pathway in the pathogenesis and progression of osteosarcoma (136).

## Final Remarks

To summarize, CXCL13 and its receptor CXCR5 have emerged as key players of cancer initiation and progression. The identification of autocrine and paracrine interactions between the tumor microenvironment and cancer cells mediated by CXCL13 highlights how autonomous and non-autonomous mechanisms contribute to the development of the cancer phenotype and the dissemination of cancer cells to metastatic sites. From our perspective, the involvement of CXCL13:CXCR5 axis in solid tumors deserves to be fully investigated. Many efforts are still required to grasp a better and more conclusive understanding of the fundamentals of this pathway. Due to the great complexities and the wide spectrum of immunological and tumoral responses in different cellular contexts, particularly considering the anti-tumorigenic vs. pro-tumorigenic actions of this pathway and the lack of specific targeting agents, it is not yet feasible to use it for therapeutic intervention in cancer patients. The elucidation of CXCR5 signaling effectors and target genes would help profiling molecular scenarios controlling tumor development and its response to targeted therapies. New discoveries in the CXCL13:CXCR5 field would also aid clinical decision-making for cancer patients, bringing us closer to the promise of translational precision medicine.

## Author Contributions

MK, MD, and MC participated in the design, writing, and editing of the manuscript, and approved it for publication. MC generated the figures for the article.

### Conflict of Interest Statement

The authors declare that the research was conducted in the absence of any commercial or financial relationships that could be construed as a potential conflict of interest.
